# An Overview of Systematic Reviews on the Effectiveness of Wenxin Keli in the Treatment of Atrial Fibrillation

**DOI:** 10.1155/2022/6973151

**Published:** 2022-05-30

**Authors:** Yuanping Wang, Yuntao Liu, Xia Yan, Dawei Wang

**Affiliations:** ^1^Shunde Hospital of Guangzhou University of Chinese Medicine, Guangzhou 528333, Guangdong, China; ^2^Guangdong Provincial Hospital of Chinese Medicine, The Second Affiliated Hospital, Guangzhou University of Chinese Medicine, Guangzhou 510120, China

## Abstract

**Background:**

Atrial fibrillation is one of the most common cardiac arrhythmias. Wenxin Keli (WXKL) is a Chinese herbal extract widely used in China to treat patients with atrial fibrillation. This study aimed to outline and summarize the current evidence of systematic reviews (SRs)/meta-analyses (MAs) investigating the clinical efficacy of WXKL in atrial fibrillation.

**Methods:**

From inception to December 2021, 6 electronic databases in English and Chinese were searched for potential SRs/MAs. The GRADE (Grading of Recommendations Assessment, Development, and Evaluation), PRISMA (Preferred Reporting Items for Systematic Reviews and Meta-analyses) checklist, and AMSTAR-2 (Assessing the Methodological Quality of Systematic Reviews-2) were used to evaluate the quality of the evidence, reporting, and methodology of SRs/MAs regarding WXKL for the treatment of atrial fibrillation.

**Results:**

A total of 8 SRs/MAs were included in the present study. The results of AMSTAR-2 and PRISMA were unsatisfactory for the main insufficiency founded in registration and protocol, search strategy, excluded study statement, evidence certainty assessment, and funding and conflict of interest information. All the included SRs/MAs were assessed as very low in methodological quality. Moreover, 23 outcomes were evaluated by GRADE for the certainty of evidence, and 2 outcomes were assessed as moderate, while 15 were low and 6 were very low. Risk of bias and publication bias contributed to the downgrading.

**Conclusion:**

WXKL may be clinically efficacious and safe for the treatment of atrial fibrillation. This finding, however, should be regarded with caution because of the low level of evidence and methodological qualities of the involved SRs/MAs. More standardized, rigorous, and comprehensive SRs/MAs and randomized control trials are needed to provide strong evidence to reach more convincing conclusions.

## 1. Introduction

Atrial fibrillation is one of the most common cardiac arrhythmias, which affects approximately 30 million individuals worldwide [1, 2]. In developed countries, the prevalence of atrial fibrillation is 1%∼4% [3], which is much higher than the prevalence in developing regions, and the incidence is expected to double in the next 20 years for the aging population [4, 5]. According to the epidemiology data, the lifetime risk of atrial fibrillation is approximately 20% in Chinese adults, and the prevalence increases with age [6]. As atrial fibrillation has a close association with other fatal diseases, cardiogenic death, heart failure, thromboembolism, and dementia, and is causing a heavy healthcare burden, it has become one of the most important public health issues [7]. The “Atrial fibrillation Better Care” (ABC) management strategy has been recommended as an integrated and holistic approach for patients with atrial fibrillation [8]. A large randomized controlled clinical trial (RCT) [9] involving 3324 subjects from 40 cities in China showed that ABC management reduced the risk of all-cause death, multiple hospitalizations, and clinical adverse outcomes. A meta-analysis [10] containing 285,000 participants also indicated that patients with atrial fibrillation following the ABC pathway had an advantage in significantly reducing all-cause mortality. However, despite progress having been made in the treatment of atrial fibrillation, the current treatment is still unsatisfactory [11]. For example, amiodarone, a widely used antiarrhythmic agent, is limited by secondary sinus bradycardia, atrioventricular block, or potentially fatal pulmonary, while radiofrequency ablation is prone to postoperative recurrence [12]. Therefore, complementary and alternative medicine may be an option for patients with atrial fibrillation; thus, more physicians in China are considering the combination of Chinese medicine with conventional treatment [13].

Wenxin Keli (WXKL), a patented Chinese medication, is extensively utilized in China as an adjuvant therapy for individuals with cardiovascular issues, particularly for the management of atrial fibrillation [14]. It is composed of *Polygonati rhizoma* (Huang Jing), *Codonopsis radix* (Dang Shen), Ambrum (Hu Po), Notoginseng Radix et Rhizoma (San Qi), and Nardostachyos Radix et Rhizoma (Gan Song). Previous studies have indicated that WXKL plays a significant role in improving the symptoms of palpitations, chest tightness, and fatigue, and has been approved as the first anti-arrhythmic Chinese medicine by the Chinese Food and Drug Administration [15]. In recent years, many scholars have focused on the mechanism explorations of WXKL in the treatment of atrial fibrillation and gained initial progress. It is reported that WXKL improves atrial remodeling in diabetic rats by regulating mitochondrial function and homeostasis [16]. What is more, results in network pharmacology indicated WXKL may regulate AF through multiple pathways—mainly in inflammatory, oxidative stress; immunization; cardiac energy metabolism; and tryptophan metabolism [17]. Further researches showed that WXKL could improve arrhythmia by modulating various ion channels, which were dominated via peak sodium current (INa) and late sodium current (INaL) [14]. In addition, WXKL can preferentially inhibit INa in atria and reduce resting membrane potential, leading to the refractory period of atrial myocytes after repolarization, and it can suppress atrial fibrillation through an anti-reentry mechanism [18]. WXKL was proved to attenuate sympathetic atrial fibrillation induced by adrenergic activation with the mechanism of modulating neurohormones by inhibiting calmodulin (CaM) expression and ryanodine receptor 2 (RYR2) phosphorylation [19]. Studies have shown that long-term oral administration of WXKL can inhibit atrial substrate remodeling caused by upregulation of Cx43, ANP, TNF-*α*, and IL-6 induced by epicardial ganglionic plexi ablation [20]. These preclinical studies suggest that WXKL is a drug with great therapeutic potential in the treatment of atrial fibrillation.

In the past decade, clinical researches on WXKL in the treatment of atrial fibrillation have also made great progress. Studies have confirmed that WXKL is a safe and effective alternative method for improving myocardial ischemia, enhancing cardiac function, alleviating ventricular remodeling, and reducing the occurrence of arrhythmias [15]. With the concept of evidence-based medicine penetrating into traditional Chinese medicine research, a growing number of systematic reviews/meta-analyses (SRs/MAs) evaluating the clinical efficacy and safety of WXKL for AF treatment are emerging [21, 22]. These SRs/MAs provided evidence-based reviews for clinical decision-making, but the evidence from SRs/MAs is currently challenged due to various risks of bias in the evidence formation process. High-quality SRs/MAs can provide reliable evidence, while low-quality SRs/MAs may instead mislead decision makers [23]. Therefore, it is necessary to summarize and analyze the quality of existing SRs/MAs on WXKL for the treatment of atrial fibrillation, to provide more comprehensive and reliable evidence for clinical decision. The present study aims to explore the quality of existing SRs/MAs systematically, proving a reasonable adjuvant treatment option for atrial fibrillation patients.

## 2. Methods

This study was registered in advance of study initiation on the website of Open Science Framework (OSF, https://osf.io/) with a registration number of DOI: 10.17605/OSF.IO/97RBP. The Cochrane Handbook guidelines and the checklist of Preferred Reporting Items for Systematic Reviews and Meta-analyses (PRISMA) were followed in the methodology [24].

### 2.1. Criteria for Study Inclusion

#### 2.1.1. Study Types

This review included SRs/MAs which discussed RCTs on WXKL for the treatment of atrial fibrillation. Studies were limited to those only published in Chinese or English. For duplicate articles, we included only the most recently published articles.

#### 2.1.2. Subject Types

According to the national or international standards, subjects diagnosed with atrial fibrillation were included regardless of sex, race, or age.

#### 2.1.3. Intervention Types

The intervention strategy in the treatment group was WXKL combined with conventional medication (e.g., warfarin, amiodarone, *β*-receptor blockers). For the control group, conventional medication was used as an intervention method.

#### 2.1.4. Outcome Types of Measurements

The outcome variables were as follows: (i) P-wave dispersion, (ii) maximum P-wave duration, (iii) maintenance rate of sinus rhythm, (iv) clinical efficiency rate, (v) left ventricular ejection fraction (LVEF), (vi) ventricular rate, (vii) recurrence rate, and (viii) adverse events.

#### 2.1.5. Search Methods for Study Identification

From inception to December 2021, we searched Wanfang, Chongqing VIP, China National Knowledge Infrastructure, PubMed, Embase, and Cochrane Library databases. Keywords for the search in these databases include atrial fibrillation, Wenxin Keli, and SRs/MAs. The PubMed search terms are shown in Table 1, and the terms were changed for different databases. To guarantee that no relevant SRs/MAs were missed, conference abstracts and the reference lists of all retrieved articles were also retrieved. The search strategy for each database is shown in Supplementary Material S1.

#### 2.1.6. Study Selection and Data Extraction

Two authors autonomously reviewed all included SRs/MAs and assessed the critical data extracted from the SRs/MAs with the studies' predefined evaluation standards. Any discrepancies among the authors were handled through discussion or by consultation with a third reviewer. The data extraction considered the first author, country, and year of publication regarding the SRs/MAs and the number of inclusion RCTs and subjects, as well as the intervention details in different groups, the outcomes that were relevant to this review, and the methods used to measure the quality of the trials. In cases of insufficient information, the study correspondence authors of the SRs/MAs were contacted by e-mail for the key data.

#### 2.1.7. Assessment of the Methodological Quality of the Included Studies

AMSTAR-2 was utilized by 2 independent authors to estimate the quality of the SRs/MAs [25]. The AMSTAR-2 is based on 16 items, 7 of which are essential (items 2, 4, 7, 9, 11, 13, and 15). The detailed list of the AMSTAR-2 can be found in Supplementary Material S2. There are three options for each assessment item: “yes,” “partial yes,” or “no.” If there is no or 1 nonessential item that does not meet the requirements, the quality is considered as “high.” If more than 1 nonessential item failed to comply, the assessment is deemed “moderate,” while when any essential item does not match, the quality is considered as “low,” and it should be assessed as very low when more than one essential item is mismatched.

#### 2.1.8. Reporting Quality Evaluation of the Included Studies

The PRISMA checklist was utilized by two independent reviewers to examine the reporting quality of the included SRs/MAs [24]. Each item on the PRISMA checklist is rated “yes,” “partial yes,” or “no” based on the degree of compliance of information supplied in the relevant studies. A detailed list can be found in Supplementary Material S3.

#### 2.1.9. Quality of Evidence

The GRADE system [26] was utilized to classify the confidence evidence of the outcomes, and the result categorized into 4 levels: very low, low, moderate, and high. The original level “high” could be reduced if the research showed publishing bias, indirectness, imprecision, or inconsistency or restrictions were identified during the evaluation. If the evidence quality is not judged as high level, this suggests that the researches are unlikely to affect the present evidence. The level “moderate” implied that future researches may have a major impact on present evidence and thus could modify the evaluation results. The two-level downgrading to “low” shows that future researches are very likely to have a major impact on the prevailing evidence and could transform the assessment conclusions. A rating of “very low” indicates that all available evidence is unidentifiable. Any differences were handled through consensus or discussed with a third reviewer who was competent and authoritative.

## 3. Results

### 3.1. Literature Search and Selection

**Figure 1:**
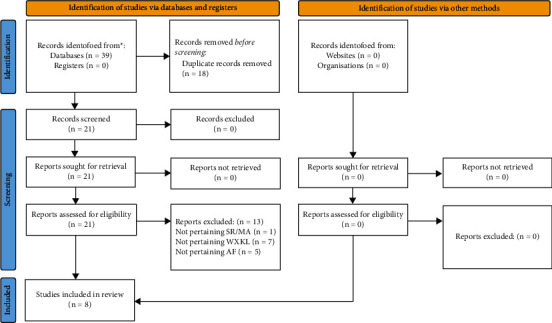
.The flowchart of the screening process.

In the initial search, 39 records were retrieved from the 6 scholarly databases based on the search strategy. After eliminating duplicates, the 21 remaining records were then screened according to titles and abstracts. Finally, only 8 [21, 22, 27–32] SRs/MAs met the requirements and were included after further full-text reading (Figure 1).

### 3.2. Characteristics of Included Systematic Reviews

The 8 SRs/MAs included in the present study were published from 2014 to 2019, all of which were published in Chinese and included 11 to 42 trials with a number of participants ranging from 805 to 4657. In the experimental group, the intervention methods were largely WXKL combined with conventional medication, and in the control group, it was conventional medication. Regarding methodological quality assessment, five SRs/MAs [27, 29–32] used the Cochrane Risk of Bias Instrument, two SRs/MAs [21, 28] used the Jadad scale, and one other SR/MA [22] used both of these scales. Details of the included SRs/MAs are described in Table 2.

### 3.3. Methodological Quality of the Included Studies

According to the guideline of the AMSTAR-2 assessment, all SRs/MAs were classified as very low methodological quality (Table 3). The methodological restriction resulted from the following reasons: firstly, none of the SRs/MAs stated the preliminary design protocol, and they also did not retrieve the potential SRs/MAs with a comprehensive search strategy. Secondly, all of the included SRs/MAs failed to provide the list of removed studies with exclusion reasons. What is more, funding information and conflict of interest were not reported in any of the 8 included SRs/Mas studies.

### 3.4. Included Studies' Reporting Quality

No study reported on every item on the PRISMA checklist (Table 4). The reporting quality of the title, abstract, introduction, and discussion was completely stated, but the major sections of the method, results, and other information in the 8 SRs/MAs were lacking. In other information sections, all of the questions (Q24∼Q27) were completely unreported (0%). In the section of methods, the reporting rates of Q7 (search strategy), Q8 (selection process), Q13f (synthesis methods), and Q15 (certainty assessment) were less than or equal to 50%. For the section of result, Q20c and Q20d in results of syntheses were described inadequately (50%), while Q22 (certainty of evidence) was completely unreported (0%) in all 8 SRs/MAs.

### 3.5. Included Studies' Evidence Quality

Table 5 summarizes the evidence quality of 23 outcomes in the 8 included SRs/MAs. The evidence quality for these outcomes was high in 0 (0/23, 0%), moderate in 2 (2/23, 8.7%), low in 15 (15/23, 65.2%), and very low in 6 (6/23, 26.1%). The downgrading of the outcomes was owing to the fact that in the included SRs/MAs of RCTs, flaws in precision, consistency, and original trials and likelihood of publication bias were detected.

## 4. Included Studies' Result Summary

### 4.1. Efficiency of WXKL for Atrial Fibrillation

#### 4.1.1. Clinical Effective Rate

*(1) WXKL* + *Conventional Medication vs*. *Conventional Medication*. Three SRs/MAs [21, 27, 31] assessed the clinical effective rate, and all results demonstrated a more significant effect in the combination therapy compared with the conventional medication (13 RCTs [21], OR = 3.40, 95% CI = 2.41 to 4.80, *P* < 0.001, *I*^2^ = 0%; 22 RCTs [31], OR = 3.37, 95% CI = 2.69 to 4.22, *P* < 0.001, *I*^2^ = 0%; 17 RCTs [27], RR = 1.22, 95% CI = 1.17 to 1.27, *P* < 0.001, *I*^2^ = 0%).

*(2) WXKL* + *Metoprolol vs*. *Metoprolol*. Two SRs/MAs [22, 29] showed that, compared with metoprolol alone, the combination with WXKL can significantly improve the clinical efficacy (10 RCTs [22], OR = 4.06, 95% CI = 2.68 to 6.15, *P* < 0.001, *I*^2^ = 4%; 4 RCTs [29], RR = 1.34, 95% CI = 1.17 to 1.54, *P* < 0.001, *I*^2^ = 13%).

*(3) WXKL* + *Amiodarone vs*. *Amiodarone*. One SR/MA [28] showed that compared with amiodarone alone, the combination with WXKL significantly improved the clinical efficacy (11 RCTs, RR = 1.22, 95% CI = 1.14 to 1.31, *P* < 0.001, *I*^2^ = 0%).

#### 4.1.2. Ventricular Rate

*(1) WXKL* + *Conventional Medication vs*. *Conventional Medication*. Three SRs/MAs suggested [21, 30, 32] that compared with conventional medication, the combination with WXKL could significantly reduce the ventricular rate (4 RCTs [21], MD = −5.86, 95% CI = −6.73 to −4.99, *P* < 0.001, *I*^2^ = 0%; 10 RCTs [32], MD = −7.14, 95% CI = −8.42 to −5.87, *P* < 0.001, *I*^2^ = 83%; 9 RCTs [30], MD = −11.66, 95% CI = −15.79 to −7.54, *P* < 0.001, *I*^2^ = 89%).

*(2) WXKL* + *Metoprolol vs*. *Metoprolol*. One SR/MA [22] showed that compared with metoprolol, the combination with WXKL could significantly reduce ventricular rate (4 RCTs, RR = −9.86, 95% CI = −17.88 to −1.84, *P* < 0.001, *I*^2^ = 91%).

#### 4.1.3. Maintenance Rate of Sinus Rhythm

*WXKL* + *Conventional Medication vs*. *Conventional Medication*. Three SRs/MAs [21, 31, 32] indicated that compared with conventional medication, the combination with WXKL could significantly improve maintenance rate of sinus rhythm (3 RCTs [21], OR = 2.76, 95% CI = 1.29 to 5.92, *P* < 0.001, *I*^2^ = 0%; 6 RCTs [32], OR = 1.19, 95% CI = 1.09 to 1.29, *P* < 0.001, *I*^2^ = 40%; 7 RCTs [31], RR = 2.32, 95% CI = 1.67 to 3.22, *P* < 0.001, *I*^2^ = 0%).

#### 4.1.4. LVEF

*(1) WXKL* + *Conventional Medication vs*. *Conventional Medication*. Two SRs/MAs [30, 32] show that compared with conventional medication, the combination with WXKL could significantly improve LVEF in patients with atrial fibrillation (4 RCTs [32], MD = 3.44, 95% CI = 0.87 to 6.01, *P* < 0.001, *I*^2^ = 54%; 9 RCTs [30], MD = 6.72, 95% CI = 4.61 to 8.84, *P* < 0.001, *I*^2^ = 65%). Significant heterogeneity was detected in the both studies; however, neither of them further explored a potential source for heterogeneity by performing subgroup analysis or sensitivity analysis.

*(2) WXKL* + *Metoprolol vs*. *Metoprolol*. One SR/MA [22] showed that compared with metoprolol, the combination with WXKL could significantly improve the LVEF value of patients with atrial fibrillation (4 RCTs, MD = 5.17, 95% CI = 3.08 to 7.26, *P* < 0.001, *I*^2^ = 13%).

#### 4.1.5. Maximum P-Wave Duration

*(1) WXKL* + *Conventional Medication vs*. *Conventional Medication*. One SR/MA [31] showed that the combination with WXKL could significantly reduce maximum P-wave duration compared with conventional medication (4 RCTs, MD = −9.91, 95% CI = −12.86 to −6.95, *P* < 0.001, *I*^2^ = 0%).

*(2) WXKL* + *Amiodarone vs*. *Amiodarone*. One SR/MA [32] showed that WXKL combined with amiodarone significantly reduced maximum P-wave duration (4 RCTs, MD = −10.75, 95% CI = −14.05 to −7.45, *P* < 0.001, *I*^2^ = 94.9%). Significant heterogeneity was detected in the study; however, no exploration was conducted for potential source of heterogeneity in the SR/MA.

#### 4.1.6. P-Wave Dispersion

*WXKL* + *Conventional Medication vs*. *Conventional Medication*. Two SRs/MAs [31, 32] showed that the combination with WXKL could significantly reduce P-wave dispersion compared with conventional medication (6 RCTs [32], MD = −4.04, 95% CI = −4.15 to −3.93, *P* < 0.001, *I*^2^ = 26%; 9 RCTs [31], MD = −5.48, 95% CI = −7.32 to −3.64, *P* < 0.001, *I*^2^ = 0%).

#### 4.1.7. Recurrence Rate

*WXKL* + *Conventional Medication vs*. *Conventional Medication*. Three SRs/MAs [27, 30, 32] showed that the recurrence rate could be significantly reduced by combining WXKL with conventional medication, and no heterogeneity was detected (5 RCTs [32], RR = 0.28, 95% CI = 0.13 to 0.59, *P* < 0.001, *I*^2^ = 0%; 2 RCTs [30], RR = 0.34, 95% CI = 0.15 to 0.76, *P* < 0.001, *I*^2^ = 0%; 4 RCTs [27], RR = 0.18, 95% CI = 0.08 to 0.41, *P* < 0.001, *I*^2^ = 0%).

### 4.2. Safety of WXKL for Atrial Fibrillation

*(1) WXKL* + *Conventional Medication vs*. *Conventional Medication*. Adverse events were reported in four SRs/MAs, with 19/34 [21], 4/10 [30], 36/79 [27], and 45/84 [32] adverse event cases in the combination group and control group, respectively. All of the adverse events were mild, such as thyroid dysfunction, gastrointestinal discomfort, bradycardia, and QT interval prolongation, which could be reversed after discontinuation of the medications.

*(2) WXKL* + *Metoprolol vs*. *Metoprolol*. Two SRs/MAs [22, 29] mentioned adverse events, and the numbers of patients with adverse events in the treatment group and the control group were 31/57 [22] and 25/23 [29], respectively, all of which were identified as mild, such as gastrointestinal reactions, dizziness, insomnia, fatigue, and other symptoms, which could be relieved by themselves after stopping the drug.

*(3) WXKL* + *Amiodarone vs*. *Amiodarone*. One SR [28] reported adverse events, and the number of patients with adverse events in the treatment group and control group was 32/69, but the description of adverse events was not reported.

## 5. Discussion

### 5.1. Summary of Main Findings

The present study assessed and provided a broad review of the efficacy and safety of WXKL in the treatment of atrial fibrillation. After a systematic search and screening, 8 SRs/MAs were included. The available evidence strongly suggested that based on conventional drugs, WXKL played an effective role in improving the clinical efficiency rate, LVEF, and maintenance rate of sinus rhythm, as well as reducing P-wave dispersion, maximum P-wave duration, ventricular rate, and recurrence rate. In addition, no severe adverse events caused by WXKL were observed during the performance of the study. However, it is important to note that the included SRs/MAs, as well as the original RCTs, were conducted with relatively poor methodological quality, and high-quality-design studies are urgently needed to further verify the evidence of its efficacy.

At present, an increasing number of clinical studies have investigated the application of WXKL as the adjunctive management strategy in the treatment of atrial fibrillation. Although these studies have obtained positive results, most SRs/MAs stated that these findings needed to be verified through multicenter, large-sample, double-blind RCTs with long-term follow-up. In addition, we evaluated the 23 outcomes of SRs/MAs using the GRADE system, and the results indicated that most of the evidence quality ranged from low to very low, with only 8.7% of the results (*n* = 2), on overall clinical effectiveness, rated moderate. The 23 outcomes were downgraded due to limitations imposed by the risk of bias in the original RCTs. We further analyzed the original RCTs by the information reported in the SRs/MAs and found that the main limitations of the original RCTs were that (1) although most of the RCTs mentioned the use of random assignment methods, the specific methods to achieve randomization were not clearly described; (2) most of the RCTs did not describe in detail how allocation concealment was performed; (3) most RCTs were not blinded to patients, investigators, or outcome analysts; and (4) none of the RCTs registered the study protocol in a publicly accessible database prior to conducting the clinical trial. In addition, the small sample size of the original RCTs, as well as the publication bias detected in the SRs/MAs, downgraded the confidence of the evidence. Herein, we recommend that researchers should strictly follow the Consolidated Standards of Reporting Trials extension for Chinese Herbal Medicine Formulas 2017 guidelines [33] when conducting RCTs evaluating WXKL in the future aiming to improve the quality of studies.

Patients with atrial fibrillation are more vulnerable to embolism and extremely prone to disability and death, making anticoagulation an important but challenging management [34] for the reason that warfarin, an anticoagulant first-line drug, and other novel anticoagulants have recognized adverse events, such as secondary bleeding, which also increase the risk of death [35]. Salvianolic acid A, a water-soluble active compound purified from *Salvia miltiorrhiza* (a major component of WXKL), has been shown to prevent acute ischemic stroke by inhibiting platelet aggregation [36] but not to increase the incidence of bleeding events [37]. Previous clinical studies [38] confirmed that WXKL in combination with warfarin significantly reduced the risk of ischemic stroke and bleeding in patients with atrial fibrillation compared with warfarin treatment. However, current evidence focusing on the anticoagulant advantages in the treatment of atrial fibrillation is still very scarce. Therefore, future research should focus on exploring the role of WXKL in anticoagulation therapy, which is essential for the treatment of atrial fibrillation.

The drug dosage is also a crucial factor affecting the clinical efficacy and safety. In the present study, only one SR/MA [31] reported the details of medication dose of the 42 original RCTs. In the limited information, most of the RCTs (88%) reported the dose was 9 g, with a frequency of 3 times per day. The dosage of WXKL is also recommended by the Chinese Pharmacopoeia 2020 edition, for the treatment of patients with atrial fibrillation. However, current SRs/MAs have insufficient evidence proving different effect in different doses of WXKL, and systematic reviewers were unable to perform subgroup analyses to select the optimal medication dose.

The present study assessed the methodological quality of SRs/MAs using the AMSTAR-2 tool, and the results showed that the eligible SRs/MAs were of low quality, with the following main items requiring improvement: (1) It is crucial to design a detailed protocol prior to the SRs/MAs, as a well-designed protocol could reduce the risk of bias during the SRs/MAs evaluation [39]. Additionally, protocol registration not only helps prevent duplication, saving time and resources, but also opens avenues for collaboration between researchers with a common interest in a topic [40]. Unfortunately, none of the SRs/MAs in this study had published protocols on publicly available websites in advance. (2) A transparent and reproducible literature screening process is necessary to avoid publication bias [41]. Nevertheless, none of the SRs/MAs in the present study provided a specific list of eliminated studies with detailed reasons for their exclusion when screening for relevant studies, which increased the risk of unreasonable exclusion, weakening the credibility and reproducibility of screening results. (3) In the literature screening and data extraction process, it is recommended that a “double abstraction process,” using two or more independent reviewers, is adopted so that any inconsistency can be negotiated or decided by a third reviewer to reduce evaluator bias. Unfortunately, some SRs/MAs did not mention the use of independent assessment by more than one reviewer during this key process. (4) None of the SRs/MAs in our study reported the source of funding of the RCTs or SRs/MAs, nor was a statement of conflict of interest provided. Previous studies [42] have indicated that SRs/MAs or RCTs that accepted funding from pharmaceutical companies were more likely to conclude with positive results.

### 5.2. Limitations

This is the first overview to thoroughly evaluate and summarize the findings of WXKL for atrial fibrillation using the PRISMA, AMSTAR-2, and GRADE review evaluation tools. This review provided methodological quality and evidence evaluation of the current SRs/MAs of WXKL as an adjunctive method for the treatment of atrial fibrillation, with a view to providing more systematic and objective evidence for decision makers and investigators. However, there are some unsatisfactory aspects that limit this study. First, due to the limitations of existing studies, all the included studies were obtained from electronic databases, and the publication languages were limited to Chinese and English, which may have induced a selection bias. Second, the RCTs included in the eligible SRs/MAs were conducted in China, and our findings may have certain regional limitations. Finally, even though we implemented a research strategy using independent-duplicate literature screening and data evaluation, the evaluation tools involved in this study are subjective, which may affect the robustness of the conclusions.

## 6. Conclusion

WXKL may be clinically efficacious and safe for the treatment of atrial fibrillation. However, the results of the present study should be interpreted with caution due to the quality of the SRs/MAs included, which were evaluated as being of low methodological and evidence quality. Standardized and rigorous RCTs and SRs/MAs conducted based on international research guidelines are needed to provide strong evidence and support convincing conclusions.

## Figures and Tables

**Table 1:** The PubMed search strategy.

#1	Atrial fibrillation [mesh]
#2	Atrial fibrillation [title/abstract]
#3	#1 OR #2
#4	WenxinKeli [mesh]
#5	WenxinKeli [title/abstract] ORWenxin [title/abstract]
#6	#4 OR #5
#7	Meta-analysis as topic [mesh]
#8	Meta-analysis [title/abstract] OR systematicreview [title/abstract] OR Cochranereview [title/abstract] OR meta-analyses
#9	#7 OR #8
#10	#3 AND #6 AND #9

**Table 2:** Features of the studies.

Author(s), year	Country	Trials (subjects)	Treatment intervention	Control intervention	Quality assessment	Main results
Du and Dai, 2014 [21]	China	13 (1050)	WXKL + CM	CM	Jadad	WXKL can increase clinical efficacy, reduce ventricular rate, improve the rate of relapse, and reduce the rate of adverse events when compared to standard CM treatment
Huang et al., 2018 [22]	China	24 (1938)	WXKL + metoprolol	Metoprolol	Cochrane criteria, Jadad	WXKL coupled with metoprolol has a higher efficacy than CM alone in the treatment of AF, and it has a decent safety profile
Li et al., 2018 [32]	China	24 (2246)	WXKL, WXKL + CM	Placebo, CM	Cochrane criteria	While WXKL alone or in combination with CM has demonstrated efficacy in the treatment of AF, this must be confirmed by high-quality data
Wang et al., 2015 [28]	China	21 (924)	WXKL + amiodarone	Amiodarone	Jadad	WXKL in combination with amiodarone has great efficacy in the treatment of AF, with few adverse effects and a generally safe side effect profile
Wang et al., 2019 [31]	China	42 (4657)	WXKL + CM	CM	Cochrane criteria	The combined application of WXKL in the treatment of AF has significant efficacy, and all main clinical efficacy indicators are superior to western anti-arrhythmic drugs alone. There is no evidence that WXKL alone can bring more benefits
Xin et al., 2019 [30]	China	11 (941)	WXKL + CM	CM	Cochrane criteria	WXKL combined with traditional CM treatment has a better effect than conventional CM treatment in lowering plasma BNP or NT-proBNP levels, slowing ventricular rate, and improving left ventricular ejection fraction in heart failure and AF patients; it has less side effects
Yang et al., 2019 [27]	China	17 (1735)	WXKL + CM	CM	Cochrane criteria	WXKL paired with CM had higher clinical effectiveness than CM alone in treating AF, with a lower recurrence rate and good safety
Zhao et al., 2014 [29]	China	11 (805)	WXKL + metoprolol	Metoprolol	Cochrane criteria	In the treatment of AF, the combination of WXKL and metoprolol was superior to metoprolol alone in terms of relieving symptoms and delaying the beginning of AF. Between the two groups, there was no significant statistical difference in the rate of adverse reactions

WXKL: Wenxin Keli; CM: conventional medication; AF: atrial fibrillation.

**Table 3:** Results of the AMSTAR-2 assessment.

Author(s), year	AMSTAR-2																Quality
	Q1	Q2	Q3	Q4	Q5	Q6	Q7	Q8	Q9	Q10	Q11	Q12	Q13	Q14	Q15	Q16	
Du et al., 2014 [21]	Y	PY	Y	PY	N	Y	N	Y	Y	N	Y	Y	Y	Y	Y	N	VL
Huang et al., 2018 [22]	Y	PY	Y	PY	N	N	N	Y	Y	N	Y	Y	Y	Y	Y	N	VL
Li et al., 2018 [32]	Y	PY	Y	PY	Y	Y	N	Y	Y	N	Y	Y	Y	Y	Y	N	VL
Wang et al., 2015 [28]	Y	PY	Y	PY	N	N	N	Y	Y	N	Y	Y	Y	Y	Y	N	VL
Wang et al., 2019 [31]	Y	PY	Y	PY	Y	Y	N	Y	Y	N	Y	Y	Y	Y	Y	N	VL
Xin et al., 2019 [30]	Y	PY	Y	PY	Y	Y	N	Y	Y	N	Y	Y	Y	Y	Y	N	VL
Yang et al., 2019 [27]	Y	PY	Y	PY	Y	Y	N	Y	Y	N	Y	Y	Y	Y	Y	N	VL
Zhao et al., 2014 [29]	Y	PY	Y	PY	N	Y	N	Y	Y	N	Y	Y	Y	Y	Y	N	VL

AMSTAR-2: Assessing the Methodological Quality of Systematic Reviews-2; Y: yes; PY: partial yes; N: no; VL: very low; L: low; M: moderate; H: high.

**Table 4:** PRISMA checklist results.

Section/topic	Items	Du et al. 2014 [21]	Huang et al. 2018 [22]	Li et al. 2018 [32]	Wang et al. 2015 [28]	Wang et al. 2019 [31]	Xin et al. 2019 [30]	Yang et al. 2019 [27]	Zhao et al. 2014 [29]	Compliance (%)
Title										
Title	1	Y	Y	Y	Y	Y	Y	Y	Y	100
Abstract										
Abstract	2	Y	Y	Y	Y	Y	Y	Y	Y	100
Introduction										
Rationale	3	Y	Y	Y	Y	Y	Y	Y	Y	100
Objectives	4	Y	Y	Y	Y	Y	Y	Y	Y	100
Methods										
Eligibility criteria	5	Y	Y	Y	Y	Y	Y	Y	Y	100
Information sources	6	Y	N	Y	Y	Y	Y	Y	Y	87.5
Search strategy	7	PY	PY	PY	PY	PY	PY	PY	PY	0
Selection process	8	PY	PY	Y	PY	Y	Y	Y	PY	50
Data collection process	9	Y	PY	Y	PY	Y	Y	Y	Y	75
Data items	10a	Y	Y	Y	Y	Y	Y	Y	Y	100
	10b	Y	Y	Y	Y	Y	Y	Y	Y	100
Study risk of bias assessment	11	PY	Y	Y	Y	Y	PY	Y	Y	75
Effect measures	12	N	Y	Y	Y	Y	Y	Y	Y	87.5
Synthesis methods	13a	Y	Y	Y	Y	Y	Y	Y	Y	100
	13b	Y	Y	Y	Y	Y	Y	Y	Y	100
	13c	Y	Y	Y	Y	Y	Y	Y	Y	100
	13d	Y	Y	Y	Y	Y	Y	Y	Y	100
	13e	N	Y	Y	Y	Y	N	Y	Y	75
	13f	N	N	Y	Y	N	N	N	N	25
Reporting bias assessment	14	Y	Y	Y	Y	Y	Y	Y	Y	100
Certainty assessment	15	N	N	N	N	N	N	N	N	0
Results										
Study selection	16a	Y	Y	Y	Y	Y	Y	Y	Y	100%
	16b	N	Y	Y	Y	Y	Y	Y	Y	87.5%
Study characteristics	17	N	Y	Y	Y	Y	Y	Y	Y	87.5%
Risk of bias in studies	18	Y	Y	Y	Y	Y	Y	Y	Y	100%
Results of individual studies	19	Y	Y	Y	Y	Y	Y	Y	Y	100%
Results of syntheses	20a	Y	Y	Y	Y	Y	Y	Y	Y	100%
	20b	Y	Y	Y	Y	Y	Y	Y	Y	100%
	20c	N	N	Y	Y	N	Y	N	Y	50%
	20d	N	N	Y	Y	N	N	Y	Y	50%
Reporting biases	21	Y	Y	Y	Y	Y	Y	Y	Y	100%
Certainty of evidence	22	N	N	N	N	N	N	N	N	0%
Discussion										
Discussion	23a	Y	Y	Y	Y	Y	Y	Y	Y	100%
	23b	Y	Y	Y	Y	Y	Y	Y	Y	100%
	23c	N	Y	Y	Y	Y	Y	Y	Y	100%
	23d	Y	Y	Y	Y	Y	Y	Y	Y	100%
Other information										
Registration and protocol	24a	N	N	N	N	N	N	N	N	0%
	24b	N	N	N	N	N	N	N	N	0%
	24c	N	N	N	N	N	N	N	N	0%
Support	25	N	N	PY	PY	PY	PY	PY	N	0%
Competing interests	26	N	N	N	N	N	N	N	N	0%
Availability of data, code, and other materials	27	N	N	N	N	N	N	N	N	0%

PRISMA: Preferred Reporting Items for Systematic Reviews and Meta-analyses; Y: yes; PY: partial yes; N: no.

**Table 5:** The certainty of evidence.

Author(s), year	Interventions	Outcomes	Trials (subjects)	Limitations	Inconsistency	Indirectness	Imprecision	Publication bias	Relative effect (95% CI)	Quality
Du et al., 2014 [21]	WXKL + CM vs. CM	Effective rate	13 (1050)	−1^①^	0	0	0	−1^⑤^	OR: 3.40 (2.41, 4.80)	Low
	WXKL + CM vs. CM	Ventricular rate	4 (319)	−1^①^	0	0	0	−1^④^	MD: −5.86 (−6.73, −4.99)	Low
	WXKL + CM vs. CM	MRSR	3 (263)	−1^①^	0	0	0	−1^④^	OR: 2.76 (1.29, 5.92)	Low
Huang et al., 2018 [22]	WXKL + metoprolol vs. metoprolol	Effective rate	10 (651)	−1^①^	0	0	−1^③^	−1^⑤^	OR: 4.06 (2.68, 6.15)	Very low
	WXKL + metoprolol vs. metoprolol	Ventricular rate	4 (263)	−1^①^	0	0	−1^③^	−1^④^	MD: −9.86 (−17.88, −1.84)	Very low
	WXKL + metoprolol vs. metoprolol	LVEF	4 (339)	−1^①^	0	0	0	−1^④^	MD: 5.17 (3.08, 7.26)	Low
Li et al., 2018 [32]	WXKL + CM vs. CM	Ventricular rate	10 (870)	−1^①^	−1^②^	0	0	0	MD: −7.14 (−8.42, −5.87)	Low
	WXKL + CM vs. CM	MRSR	6 (648)	−1^①^	0	0	0	−1^④^	RR: 1.19 (1.09, 1.29)	Low
	WXKL + CM vs. CM	Recurrence rate	5 (346)	−1^①^	0	0	0	−1^④^	RR: 0.28 (0.13, 0.59)	Low
	WXKL + CM vs. CM	LVEF	4 (402)	−1^①^	−1^②^	0	−1^③^	−1^④^	MD: 3.44 (0.87, 6.01)	Very low
	WXKL + amiodarone vs. amiodarone	Pmax	4 (388)	−1^①^	−1^②^	0	0	−1^④^	MD: −10.75 (−14.05, −7.45)	Very low
	WXKL + CM vs. CM	Pd	6 (603)	−1^①^	0	0	0	−1^④^	MD: −4.04 (−4.15, −3.93)	Low
Wang et al., 2015 [28]	WXKL + amiodarone vs. amiodarone	Effective rate	11 (854)	−1^①^	0	0	0	0	RR: 1.22 (1.14, 1.31)	Moderate
Wang et al., 2019 [31]	WXKL + CM vs. CM	Effective rate	22 (2328)	−1^①^	0	0	0	0	OR: 3.37 (2.69, 4.22)	Moderate
	WXKL + CM vs. CM	MRSR	7 (856)	−1^①^	0	0	0	−1^④^	OR: 2.32 (1.67, 3.22)	Low
	WXKL + CM vs. CM	Pmax	4 (319)	−1^①^	0	0	0	−1^④^	MD: −9.91 (−12.86, −6.95)	Low
	WXKL + CM vs. CM	Pd	9 (732)	−1^①^	0	0	0	−1^④^	MD: −5.48 (−7.32, −3.64)	Low
Xin et al., 2019 [30]	WXKL + CM vs. CM	Ventricular rate	9 (632)	−1^①^	−1^②^	0	0	−1^④^	MD: −11.66 (−15.79, 7.54)	Very low
	WXKL + CM vs. CM	Recurrence rate	2 (184)	−1^①^	0	0	0	−1^④^	RR: 0.34 (0.15, 0.76)	Low
	WXKL + CM vs. CM	LVEF	9 (694)	−1^①^	−1^②^	0	0	−1^④^	MD: 6.72 (4.61, 8.84)	Very low
Yang et al., 2019 [27]	WXKL + CM vs. CM	Effective rate	17 (1735)	−1^①^	0	0	0	−1^⑤^	RR: 1.22 (1.17, 1.27)	Low
	WXKL + CM vs. CM	Recurrence rate	4 (353)	−1^①^	0	0	0	−1^④^	RR: 0.18 (0.08, 0.41)	Low
Zhao et al., 2014 [29]	WXKL + metoprolol vs. metoprolol	Effective rate	4 (269)	−1^①^	0	0	0	−1^④^	RR: 1.34 (1.17, 1.54)	Low

GRADE: Grading of Recommendations Assessment, Development, and Evaluation; OR: odds ratio; RR: relative risk; MD: mean difference; VL: very low; L: low; H: high; MRSR: maintenance rate of sinus rhythm; CM: conventional medication. ①: the experimental design had a large bias in random, distributive findings or was blind. ②: the confidence intervals overlapped less, the *P* value for the heterogeneity test was very small, and the *I*^2^ was larger. ③: the confidence interval was not narrow enough. ④: fewer studies were included, and there may have been greater publication bias. ⑤: funnel graph asymmetry.

## Data Availability

The data used to support the findings of this study are available from the corresponding authors upon request.

## Abbreviations

GRADE: Grading of Recommendations Assessment, Development, and Evaluation
PRISMA: Preferred Reporting Items for Systematic Reviews and Meta-analyses
AMSTAR-2: Assessing the Methodological Quality of Systematic Reviews-2
WXKL: Wenxin Keli
SRs/MAs: Systematic reviews/meta-analyses
RCTs: Randomized controlled trials
LVEF: Left ventricular ejection fraction.

## Supplementary Materials

Supplementary Materials Supplementary Material S1. The search strategy for each database. Supplementary Material S2. The 16 items of AMSTAR-2. Supplementary Material S3. The 27 items of PRISMA.
